# Entanglement concentration for arbitrary four-particle linear cluster states

**DOI:** 10.1038/s41598-017-02146-9

**Published:** 2017-05-16

**Authors:** Ting-Ting Song, Xiaoqing Tan, Tianyin Wang

**Affiliations:** 1 Department of Computer Science, College of Information Science and Technology, Jinan University, Guangzhou, 510632 China; 2 Department of Mathematics, College of Information Science and Technology, Jinan University, Guangzhou, 510632 China; 3 School of Mathematical Science, Luoyang Normal University, Luoyang, 471022 China

## Abstract

Cluster states, whose model are a remarkably rich structure in measurement-based quantum computation, hold high degree of entanglement, while entanglement is very fragile during the process of transmission because of the inevitable interaction with the environment. We propose two entanglement concentration protocols for four-particle linear cluster states which and are susceptible to the decoherence and the imperfect communication setups. In the first protocol, POVM operators are introduced to maximize the success probability, and the second protocol is based on cross-Kerr nonlinearity which is utilized to check the parity between the original particle and the ancillary particle. Both of the protocols have their own advantages. The first one can be easily realized in experiment by linear optics, while the one with cross-Kerr nonlinearity reach more than 90% success probability by iteration. Since the wide application of cluster states, the two protocols are efficient and valuable to different fields of quantum communication.

## Introduction

Entanglement, the genuine quantum phenomenon in quantum world, is not equivalent to the analog of classical physical theory. It is therefore of importance to explore quantum states with entanglement. Cluster states^1, 2^, one special kind of entangled states, develop one of two kinds of quantum computation, the so-called measurement-based quantum computation^3, 4^, while the other one is quantum circuit model of quantum computation. The one-dimension *N*-particle cluster states are also called linear cluster states (LCSs)^1^. When *N* = 2, the two-particle LCSs are local unitary equivalent to Bell states. The three-particle LCSs are local unitary equivalent to GHZ states, but four-particle LCSs are not local unitary equivalent to four-particle GHZ states. Compared with GHZ class and W class, the four-particle LCSs hold high degree of entanglement. Besides the theoretical research, Cluster states have already been realized experimentally^1, 5, 6^, like in optical lattices of cold atoms with Ising type interactions^1^. In 2004, Nielsen^7^ proposed an approach to prepare cluster states with non-deterministic quantum gates. Then, Browne and Rudolph^8^ introduced two-dimensional array of qubits into the preparation of cluster states. A four-particle LCS^9^ and a six-atom ‘Schrodinger cat’ state^10^ were already achieved in experiment.

Besides the measurement-based quantum computation, many quantum communication protocols are also based on the entanglement principle in cluster states, such as quantum error-correction codes^11^, quantum dense coding^12, 13^, quantum information splitting^14^, quantum teleportation^12, 13^, quantum key distribution^15, 16^, quantum secret sharing^13, 17, 18^, quantum secure direct communication^19, 20^. However, the entanglement is very fragile during the process of transmission because of the inevitable interaction with the environment. Affected by the decoherence in transmission channels^21^, the fidelity of the entangled cluster states degrades. Two quantum techniques, entanglement purification^22–26^ and entanglement concentration^27–34^, are introduced to improve the fidelity of the entangled particle system. In detailed, entanglement purification distills mixed states into a perfect entangled state, while entanglement concentration protocols (ECPs) are used to obtain a perfect pure state from some partially entangled pure states. In 1996, Bennett *et al*.^27^ introduced an ECP based on Schimidt projection method. Later, Bose *et al*.^28^ proposed an ECP with the entanglement swapping. Then other two entanglement concentration protocols were proposed^29, 30^. These schemes distilled the perfect entangled states from some partial entangled states with linear optics. If the entanglement concentration operations are nonlinear, refs 31 and 32 are concerned on the topic. In 2012, Sheng *et al*.^33^ presented two ECPs for arbitrary three-particle W states that exploit linear optics and cross-Kerr nonlinearity separately, and showed that the latter ECP can reach higher success probability by iterating some steps many times. Later, they^34^ did the same project using quantum-dot and optical microcavities under the single-particle assistance.

Owing the high degree of entanglement, four-particle LCSs have wide applications in quantum communication protocols^12, 14, 19, 20^. Because of the high degree of entanglement, it is more difficult to concentrate the entanglement of four-particle LCSs via traditional methods. The weak nonlinearity concepts^35, 36^ have been introduced to the area of quantum computation^37, 38^, the distribution of entanglement^39, 40^, and the generation of cluster states^41, 42^ where successful probabilities for the generation of three-qubit states are much efficient. Based on the new concept, ref. 43 proposed a way to concentrate the entanglement of four-particle LCSs. However, it is hard to applied in practices because of the three times parity-checking and Toffoli gate. The present paper proposes two ways of entanglement concentration for four-particle LCSs, one with linear optics, the other one based on the weak cross-Kerr nonlinearity. Different from the ECPs for GHZ class and W class via linear optics and cross-Kerr nonlinearity, the ways of present ECPs are much novel. In the first protocol, POVM operators are introduced to maximize the success probability, and the second protocol is based on cross-Kerr nonlinearity which is utilized to check the parity between the original particle and the ancillary particle. Both of the protocols have their own advantages. The first one can be easily realized in experiment by linear optics, while the one with cross-Kerr nonlinearity can reach more than 90% successful probability by iteration.

## Results

Alice wants to share a four-particle LCS, defined as

1 $${|\psi \rangle }_{1234}=\frac{1}{2}{(|HHHH\rangle +|HHVV\rangle +|VVHH\rangle -|VVVV\rangle )}_{1234}$$

through quantum channels with Bob, Charlie and Daniel. Affected by the decoherence of entanglement arising from the storage process or the imperfect entanglement source, the entanglement of four-particle LCS decreases. Now we consider the case that if the state after transmitted is

2 $${|{\rm{\Psi }}\rangle }_{1234}={\lambda }_{0}{|HHHH\rangle }_{1234}+{\lambda }_{1}{|HHVV\rangle }_{1234}+{\lambda }_{2}{|VVHH\rangle }_{1234}+{\lambda }_{3}{|VVVV\rangle }_{1234}\mathrm{.}$$

Here the subscripts 1, 2, 3, 4 means the four particles kept by Alice, Bob, Charlie and Daniel respectively (The concentration of entanglement on other pure states is beyond the reach of the following two ways, and that will be the future work). Four real parameters *λ*_*i*_ ≠ 0(*i* = 0, 1, 2, 3) known to four parties satisfy the normalization condition |*λ*_0_|^2^ + |*λ*_1_|^2^ + |*λ*_2_|^2^ + |*λ*_3_|^2^ = 1. Without loss of generality, suppose |*λ*_0_| ≤ |*λ*_1_| and |*λ*_0_| ≤ |*λ*_2_|. If |*λ*_0_| is bigger than |*λ*_1_| or |*λ*_2_|, by performing local unitary operations, four parties can always make the absolute value of the coefficient of |*HHHH*〉_1234_ smaller than |*λ*_1_| and |*λ*_2_|. The two ways of entanglement concentration for four-particle LCSs are as follows.

### ECP with linear optics

In order to distill perfect cluster state by linear optics from the state in Eq. (2), local operations are needed. Any local POVM operations performed on particle *i* is as

3 $${L}_{i}={a}_{i}|H\rangle \langle H|+{b}_{i}|H\rangle \langle V|+{c}_{i}|V\rangle \langle H|+{d}_{i}|V\rangle \langle V|,\,i=1,2,3,4,$$

where *a*_*i*_, *b*_*i*_, *c*_*i*_ and *d*_*i*_ are all real number and satisfy |*a*_*i*_| + |*c*_*i*_| ≤ 1 and |*b*_*i*_| + |*d*_*i*_| ≤ 1. If Alice, Bob, Charlie and Daniel are only permitted to perform local operations on the particles hold by themselves, the state |Ψ〉_1234_ will be changed into *L*_1_ ⊗ *L*_2_ ⊗ *L*_3_ ⊗ *L*_4_|Ψ〉_1234_. After four local POVM operations, the ECP for four-particle LCS with linear optics is finished, which means that the whole system hold by four parties is hoped to be the cluster state in Eq. (1). Thus the coefficients of terms in the final state should satisfy the following basic conditions,

4 $${f}_{1}={f}_{4}={f}_{13}=-\,{f}_{16}\ne \mathrm{0,}\,{f}_{i}=0,i\in {\mathrm{[1},\mathrm{16]}}_{Z}\backslash \mathrm{\{1},4,13,\mathrm{16\},}$$

where the coefficient of *i*th term is denoted as *f*_*i*_. According to the conditions, four parties can find the relationship between *a*_*i*_, *b*_*i*_, *c*_*i*_, *d*_*i*_(*i* = 1, 2, 3, 4) and *λ*_*i*_(*i* = 1, 2, 3, 4), then they would know the detailed operations performed by themselves to concentrate the imperfect cluster state.

In order to describe the process of ECP with linear optics clearly, we consider a special case, when there is no operation on particle 4, i.e. *L*_4_ = *I* = |*H*〉〈*H*| + |*V*〉〈*V*| which means *a*_4_ = *d*_4_ = 1 and *b*_4_ = *c*_4_ = 0. We show how to find the unknown parameters in the other three POVMs those can concentrate the entanglement in |Ψ〉_1234_ of Eq. (2) with the maximum success probability. The final system without normalized is changed into |Ψ′〉_1234_ = *L*_1_ ⊗ *L*_2_ ⊗ *L*_3_ ⊗ *I*|Ψ〉_1234_, which also has the form of cluster state in Eq. (1) after normalized. If the coefficients are denoted as *f*′(*i*), *i* ∈ [1, 16]_*Z*_, they satisfy the conditions

5 $${f^{\prime} }_{1}={f^{\prime} }_{4}={f^{\prime} }_{13}=-{f^{\prime} }_{16}\ne \mathrm{0,}\,{f^{\prime} }_{i}=0,i\in {\mathrm{[1},\mathrm{16]}}_{Z}\backslash \mathrm{\{1},4,13,\mathrm{16\}.}$$

The success probability that four parties transform |Ψ〉_1234_ of Eq. (2) into |*ψ*〉_1234_ of Eq. (1) is *P* = |*f*′_1_|^2^ + |*f*′_4_|^2^ + |*f*′_13_|^2^ + |*f*′_16_|^2^ = 4|*f*′_1_|^2^. Now our aim to concentrate the arbitrary four-particle cluster states can be divided into two steps. The first step is to solve the parameters *a*_*i*_, *b*_*i*_, *c*_*i*_, *d*_*i*_ (*i* = 1, 2, 3) with respect to *λ*_*i*_(0 ≤ *i* ≤ 3) according to the conditions in Eq. (5). The second step is to maximize the success probability *P*. We solve them one by one as follows.

There are three kinds of relationship satisfying the conditions in Eq. (5), and the detailed process is shown in Supplementary Material. The first solution is *λ*_1_ *λ*_2_ − *λ*_0_ *λ*_3_ = 0. The second kind is

6 $$\{\begin{array}{l}{a}_{1}={d}_{1}={a}_{2}={d}_{2}={b}_{3}={c}_{3}=\mathrm{0,}\\ {b}_{1}{c}_{1}{b}_{2}{c}_{2}{a}_{3}{d}_{3}\ne \mathrm{0,}\\ {\lambda }_{2}{a}_{3}={\lambda }_{3}{d}_{3},\\ {\lambda }_{2}{b}_{1}{b}_{2}={\lambda }_{0}{c}_{1}{c}_{2},\\ {\lambda }_{1}{\lambda }_{2}+{\lambda }_{0}{\lambda }_{3}=\mathrm{0,}\end{array}$$

and the third solution is

7 $$\{\begin{array}{l}{b}_{1}={c}_{1}={b}_{2}={c}_{2}={b}_{3}={c}_{3}=\mathrm{0,}\\ {a}_{1}{d}_{1}{a}_{2}{d}_{2}{a}_{3}{d}_{3}\ne \mathrm{0,}\\ {\lambda }_{2}{a}_{3}+{\lambda }_{3}{d}_{3}=\mathrm{0,}\\ {\lambda }_{1}{a}_{1}{a}_{2}+{\lambda }_{3}{d}_{1}{d}_{2}=\mathrm{0,}\\ {\lambda }_{1}{\lambda }_{2}+{\lambda }_{0}{\lambda }_{3}=0.\end{array}$$

Secondly, following the three solutions of the relationship between *λ*_*i*_ and the coefficients of *L*_*i*_, we maximize the success probability of each solution to obtain the detailed POVMs operators. The maximization of the local probabilities

8 $$\{\begin{array}{l}\det (I-{L}_{1}^{\dagger }{L}_{1})=\mathrm{0,}\\ \det (I-{L}_{2}^{\dagger }{L}_{2})=\mathrm{0,}\\ \det (I-{L}_{3}^{\dagger }{L}_{3})=\mathrm{0,}\end{array}$$

implies that the constraints

9 $$\{\begin{array}{l}\mathrm{(1}-|{b}_{1}{|}^{2}\mathrm{)(1}-|{c}_{1}{|}^{2})=\mathrm{0,}\\ \mathrm{(1}-|{b}_{2}{|}^{2}\mathrm{)(1}-|{c}_{2}{|}^{2})=\mathrm{0,}\\ \mathrm{(1}-|{a}_{3}{|}^{2}\mathrm{)(1}-|{d}_{3}{|}^{2})=\mathrm{0,}\end{array}$$

should be satisfied and that the state is transformed into the cluster state with the maximized success probability. The first solution with less constraints is hard to obtain the particular POVMs, so we take the second solution as an example to show how to maximize the success probability (Actually, the maximization of the success probability with the third solution is similar with that with the second solution.). Under the conditions in Eqs (6) and (9), the parameters with

$$|{b}_{1}|=|{c}_{1}|=|{c}_{2}|=|{a}_{3}|=1,\,{b}_{2}=\frac{{\lambda }_{0}{c}_{1}{c}_{2}}{{\lambda }_{2}{b}_{1}},{d}_{3}=\frac{-{\lambda }_{0}{a}_{3}}{{\lambda }_{1}}$$

or

$$|{b}_{2}|=|{c}_{2}|=|{c}_{1}|=|{a}_{3}|=1,{b}_{1}=\frac{{\lambda }_{0}{c}_{1}{c}_{2}}{{\lambda }_{2}{b}_{2}},{d}_{3}=\frac{-{\lambda }_{0}{a}_{3}}{{\lambda }_{1}}$$

make the success probability maximum as 4|*λ*_0_|^2^. One solution for the local POVM operations on three particles is

10 $${L}_{1}=|H\rangle \langle V|+|V\rangle \langle H|,{L}_{2}=\frac{{\lambda }_{0}}{{\lambda }_{2}}|H\rangle \langle V|+|V\rangle \langle H|,{L}_{3}=|H\rangle \langle H|-\frac{{\lambda }_{0}}{{\lambda }_{1}}|V\rangle \langle V|,{L}_{4}=I,$$

with the success probability *P* = 4|*λ*_0_|^2^, if there exists *λ*_0_ *λ*_3 _= −*λ*_1_ *λ*_2_. That means if Daniel doesn’t perform any operations, Alice, Bob and Charlie can concentrate the entanglement of |Ψ〉_1234_ into |*ψ*〉_1234_ with the success probability *P* = 4|*λ*_0_|^2^ by performing the local operations *L*_1_, *L*_2_ and *L*_3_ in Eq. (10) respectively.

Furthermore, in the cluster state, particle 1 is symmetric with particle 2, so the solutions for maximizing success probability can be interchanged over particle 1 and particle 2. At the same time, particle 3 is symmetric with particle 4, thus the POVM operations over particle 3 and particle 4 can also be interchanged. Considering the symmetry over particle 1 and particle 2 (particle 3 and particle 4), only two parties from four perform local POVMs and distill the cluster state in Eq. (2) into |*ψ*〉_1234_. As the process that obtaining the parameters in three local POVMs, we can get one of solutions in the case is

11 $${L}_{1}=I,{L}_{2}=|H\rangle \langle H|-\frac{{\lambda }_{1}}{{\lambda }_{3}}|V\rangle \langle V|,{L}_{3}=|H\rangle \langle H|+\frac{{\lambda }_{0}}{{\lambda }_{1}}|V\rangle \langle V|,\,{L}_{4}=I,$$

with the success probability *P* = 4|*λ*_0_|^2^, if *λ*_0_ *λ*_3_ = −*λ*_1_ *λ*_2_ exists. Thus, by performing the operations in Eq. (11), Alice, Bob, Charlie and Daniel also can distill perfect cluster state from |Ψ〉_1234_ with the success probability *P* = 4|*λ*_0_|^2^.

**Figure 1:**
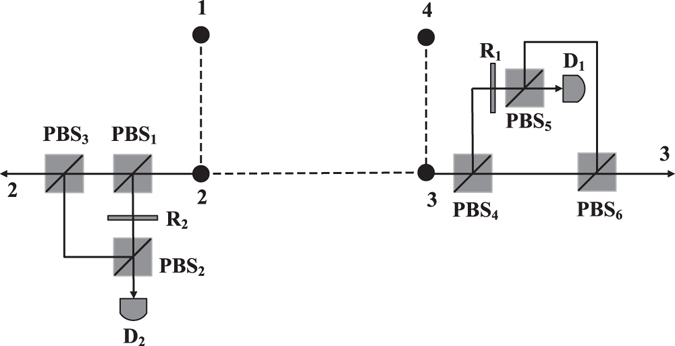
Schematic drawing of ECP for a four-particle cluster state with linear optics. PBS represents a polarizing beam splitter, which transmits the particle in the horizontal polarization |*H*〉 and reflects the particle in the vertical polarization |*V*〉. *R*_*i*_ represents a wave plate which can rotate the vertical polarization |*V*〉 with an angle *θ*_*i*_ = arccos (*λ*_0_/*λ*_*i*_). Symbol *D*_1_ and *D*_2_ are the single-photon detectors.

All the solutions of the ECP can be implemented by linear optics, polarization beam splitter and rotated operations, which is practical and economical. We take the solution in Eq. (11) as an example to show how to implement an ECP by linear optics. The schematic drawing is as Fig. 1.

According to the principle of our last entanglement concentration protocol with *λ*_1_ *λ*_2_ = −*λ*_0_ *λ*_3_, only particle 2 and particle 3 are operated by some local POVM operations. After through *PBS*_1_ (*PBS*_4_), the vertical component in particle 2 (3) is rotated by *R*_2_ (*R*_1_). The wave plate *R*_*i*_ is used to rotate |*V*〉 with an angle *θ*_*i*_ = arccos (*λ*_0_/*λ*_*i*_), that is $|V\rangle \to \sqrt{| {\lambda }_{i}{| }^{2}-| {\lambda }_{0}{| }^{2}}/{\lambda }_{i}|H\rangle +{\lambda }_{i}|V\rangle$. After the vertical component of particle 2 and that of particle 3 pass through wave plates *R*_2_ and *R*_1_, respectively, the state in Eq. (2) is changed into

12 $$\begin{array}{rcl}{|{\rm{\Phi }}\rangle }_{1234} & = & {\lambda }_{0}{|HHHH\rangle }_{1234}+{|HHV\rangle }_{124}(\sqrt{|{\lambda }_{1}{|}^{2}-|{\lambda }_{0}{|}^{2}}{|H\rangle }_{3}+{\lambda }_{0}{|V\rangle }_{3})\\  &  & +{|VHH\rangle }_{134}\otimes (\sqrt{|{\lambda }_{2}{|}^{2}-|{\lambda }_{0}{|}^{2}}{|H\rangle }_{2}+{\lambda }_{0}{|V\rangle }_{2})\\  &  & +\frac{1}{{\lambda }_{0}}{|VV\rangle }_{14}(\sqrt{|{\lambda }_{2}{|}^{2}-|{\lambda }_{0}{|}^{2}}{|H\rangle }_{2}+{\lambda }_{0}{|V\rangle }_{2})\\  & \otimes  & (\sqrt{|{\lambda }_{1}{|}^{2}-|{\lambda }_{0}{|}^{2}}{|H\rangle }_{3}+{\lambda }_{0}{|V\rangle }_{3})\mathrm{.}\end{array}$$

Then the vertical component of particle 2 and that of particle 3 in |Φ〉_1234_ are reflected by *PBS*_2_ and *PBS*_5_, respectively, while both of the horizontal components arrive the detectors. In theory, Alice can judge the protocol succeeds or not, according to the response of detectors. If particle 2 or particle 3 reaches detector *D*_2_ or *D*_1_, the ECP protocol fails. When particle 2 and particle 3 pass through *PBS*_2_, *PBS*_3_, *PBS*_5_ and *PBS*_6_, the whole state is transformed into |*ψ*〉_1234_ in Eq. (1) with the success probability 4|*λ*_0_|^2^.

### ECP with cross-Kerr nonlinearity

**Figure 2:**
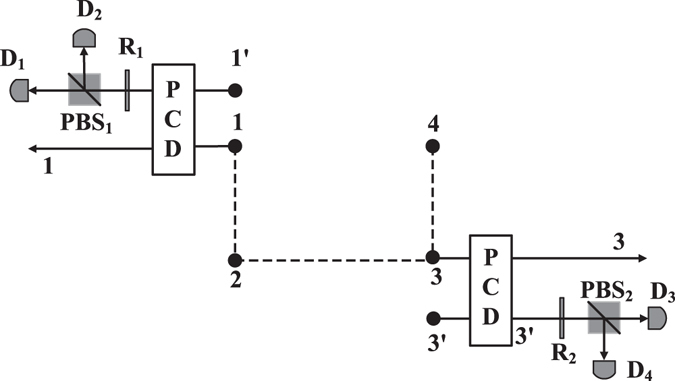
Schematic drawing of ECP for a four-particle cluster state with ancillary particles. PCD means the “parity checking device” which distinguishes the parity between particle *i* and ancillary particle *i*′ using cross-Kerr nonlinearity. *PBS*_*i*_ represents a polarizing beam splitter, which transmits the particle in the horizontal polarization |*H*〉 and reflects the particle in the vertical polarization |*V*〉. *R*_*i*_ represents a wave plate which represents a Hadamard operation on the ancillary single particle.

The section introduces the other way to concentrate the entanglement of the four-particle state in Eq. (2) with cross-Kerr nonlinearity^35, 36^, which is based on the quantum non-demolition detection. The ECP for four-particle cluster state with cross-Kerr nonlinearity improves the success probability by iteration. The principle is shown in Fig. 2.

Suppose Alice, Bob, Charlie and Daniel hold the particles 1, 2, 3 and 4 respectively. Firstly, Alice prepares an ancillary particle 1′ in ${|\varphi \rangle }_{1^{\prime} }=\frac{1}{\sqrt{| {\lambda }_{1}{| }^{2}+| {\lambda }_{3}{| }^{2}}}({\lambda }_{3}|{H}\rangle -{\lambda }_{1}|{V}\rangle )$, so the whole system is

13 $$\begin{array}{rcl}{|{\rm{\Psi }}\rangle }_{1234}\otimes {|\varphi \rangle }_{1^{\prime} } & = & [{\lambda }_{1}{|HH\rangle }_{12}(\frac{{\lambda }_{0}}{{\lambda }_{1}}{|HH\rangle }_{34}+{|VV\rangle }_{34})+{\lambda }_{3}{|VV\rangle }_{12}\\  & \otimes  & (\frac{{\lambda }_{2}}{{\lambda }_{3}}{|HH\rangle }_{34}+{|VV\rangle }_{34})]\otimes \frac{{\lambda }_{3}{|H\rangle }_{1^{\prime} }-{\lambda }_{1}{|V\rangle }_{1^{\prime} }}{\sqrt{|{\lambda }_{1}{|}^{2}+|{\lambda }_{3}{|}^{2}}}\\  & = & [{\lambda }_{1}{|HH\rangle }_{12}(\frac{{\lambda }_{0}}{{\lambda }_{1}}{|HH\rangle }_{34}+{|VV\rangle }_{34})-{\lambda }_{3}{|VV\rangle }_{12}\\  & \otimes  & (\frac{{\lambda }_{0}}{{\lambda }_{1}}{|HH\rangle }_{34}-{|VV\rangle }_{34})]\otimes \frac{{\lambda }_{3}{|H\rangle }_{1^{\prime} }-{\lambda }_{1}{|V\rangle }_{1^{\prime} }}{\sqrt{|{\lambda }_{1}{|}^{2}+|{\lambda }_{3}{|}^{2}}}\\  & = & \frac{{\lambda }_{1}{\lambda }_{3}}{\sqrt{|{\lambda }_{1}{|}^{2}+|{\lambda }_{3}{|}^{2}}}[{|HHH\rangle }_{11^{\prime} 2}{(\frac{{\lambda }_{0}}{{\lambda }_{1}}|HH\rangle +|VV\rangle )}_{34}\\  &  & +{|VVV\rangle }_{11^{\prime} 2}{(\frac{{\lambda }_{0}}{{\lambda }_{1}}|HH\rangle -|VV\rangle )}_{34}]\\  &  & -\frac{{\lambda }_{1}^{2}}{\sqrt{|{\lambda }_{1}{|}^{2}+|{\lambda }_{3}{|}^{2}}}{|HVH\rangle }_{11^{\prime} 2}{(\frac{{\lambda }_{0}}{{\lambda }_{1}}|HH\rangle +|VV\rangle )}_{34}\\  &  & -\frac{{\lambda }_{3}^{2}}{\sqrt{|{\lambda }_{1}{|}^{2}+|{\lambda }_{3}{|}^{2}}}{|VHV\rangle }_{11^{\prime} 2}{(\frac{{\lambda }_{0}}{{\lambda }_{1}}|HH\rangle -|VV\rangle )}_{34},\end{array}$$

where *λ*_0_ *λ*_3 _= −*λ*_1_ *λ*_2_ is applied in the second equation.

**Figure 3:**
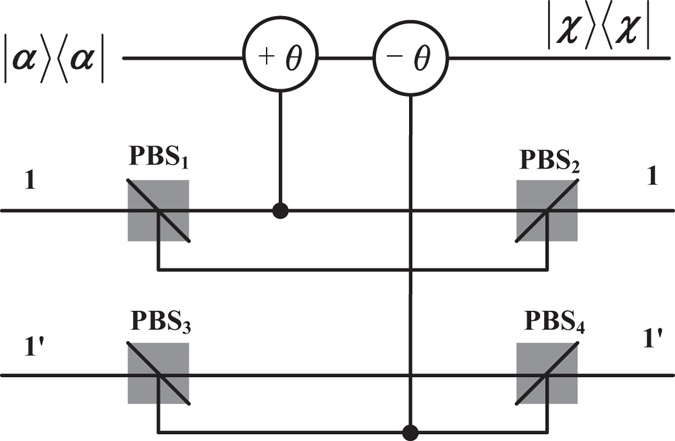
Schematic drawing of PCD operated on the original particle 1 and the ancillary particle 1′^35^. ±*θ* represents that cross-Kerr nonlinearity makes |*α*〉 into |*αe* ^±*iθ*^〉 when there is a particle passing. The even-parity states |*HH*〉 and |*VV*〉 will introduce phase shift ±*θ* to |*α*〉, while the odd-parity states |*HV*〉 and |*VH*〉 result in no phase shift. |*χ*〉〈*χ*| is the homodyne measurement that can distinguish different phase shifts.

Based on the setups in Fig. 3 ^35^, Alice checks the parity on particle 1 and particle 1′, and measures the particle 1′ in the diagonal basis $|\pm \rangle =\frac{1}{\sqrt{2}}(|H\rangle \pm |V\rangle )$. If the measurement result is |+〉 (|−〉), Alice operates *I* (*σ*_*Z*_ = |*H*〉〈*H*| − |*V*〉〈*V*|) on particle 1. Then according to the output of PCD (parity checking device), the system is divided into two classes. After Alice’s operations, the normalized system with even parity is

14 $$\begin{array}{c}\begin{array}{rcl}{|\varphi \rangle }_{1234}^{A1e} & = & \frac{1}{\sqrt{2({|\frac{{\lambda }_{0}}{{\lambda }_{1}}|}^{2}+1)}}[{|HH\rangle }_{12}{(\frac{{\lambda }_{0}}{{\lambda }_{1}}|HH\rangle +|VV\rangle )}_{34}\\  &  & +\,{|VV\rangle }_{12}{(\frac{{\lambda }_{0}}{{\lambda }_{1}}|HH\rangle -|VV\rangle )}_{34}]\\  & = & \frac{1}{\sqrt{2({|\frac{{\lambda }_{0}}{{\lambda }_{1}}|}^{2}+1)}}(\frac{{\lambda }_{0}}{{\lambda }_{1}}{|HH\rangle }_{34}{(|HH\rangle +|VV\rangle )}_{12}+{|VV\rangle }_{34}{(|HH\rangle -|VV\rangle )}_{12})\end{array}\end{array}$$

with the probability ${P}_{A1}^{e}=\frac{2{|{\lambda }_{1}{\lambda }_{3}|}^{2}}{{|{\lambda }_{1}|}^{2}+{|{\lambda }_{3}|}^{2}}({|\frac{{\lambda }_{0}}{{\lambda }_{1}}|}^{2}+1)$, and the normalized system with odd parity is

15 $$\begin{array}{rcl}{|\varphi \rangle }_{1234}^{A1o} & = & \frac{{\lambda }_{1}^{2}}{\sqrt{{P}_{A1}^{o}(|{\lambda }_{1}{|}^{2}+|{\lambda }_{3}{|}^{2})}}{|HH\rangle }_{12}{(\frac{{\lambda }_{0}}{{\lambda }_{1}}|HH\rangle +|VV\rangle )}_{34}\\  &  & +\frac{{\lambda }_{3}^{2}}{\sqrt{{P}_{A1}^{o}(|{\lambda }_{1}{|}^{2}+|{\lambda }_{3}{|}^{2})}}{|VV\rangle }_{12}{(\frac{{\lambda }_{0}}{{\lambda }_{1}}|HH\rangle -|VV\rangle )}_{34}\end{array}$$

with the probability ${P}_{A1}^{o}=\frac{{|{\lambda }_{1}|}^{4}+{|{\lambda }_{3}|}^{4}}{{|{\lambda }_{1}|}^{2}+{|{\lambda }_{3}|}^{2}}({|\frac{{\lambda }_{0}}{{\lambda }_{1}}|}^{2}+1)$.

Compared the coefficients of $|\varphi {\rangle }_{1234}^{A{\rm{1e}}}$ with those of $|\varphi {\rangle }_{1234}^{A1o}$, the first two coefficients in $|\varphi {\rangle }_{1234}^{A{\rm{1e}}}$ are same (so are the last two coefficients), while all the coefficients in $|\varphi {\rangle }_{1234}^{A1o}$ are different, similar with the property of the initial state |Ψ〉_1234_. If the state is in $|\varphi {\rangle }_{1234}^{A{\rm{1e}}}$, Alice’s step is successful, then Alice tells Charlie to continue to perform the following steps of the protocol. When the system is in state $|\varphi {\rangle }_{1234}^{A1o}$, Alice fails and needs to do the above operations again.

If Alice fails, according to Eq. (15) she prepares another ancillary particle with the form

16 $${|\varphi \rangle }_{1^{\prime\prime} }=\frac{{\lambda }_{3}^{2}}{\sqrt{|{\lambda }_{1}{|}^{4}+|{\lambda }_{3}{|}^{4}}}|H\rangle +\frac{{\lambda }_{1}^{2}}{\sqrt{|{\lambda }_{1}{|}^{4}+|{\lambda }_{3}{|}^{4}}}|V\rangle \mathrm{.}$$

The total system is in

17 $$\begin{array}{c}\begin{array}{rcl}{|\varphi \rangle }_{1234}^{A1o}\otimes {|\varphi \rangle }_{1^{\prime\prime} } & = & [\frac{{\lambda }_{1}^{2}}{\sqrt{{P}_{A1}^{o}(|{\lambda }_{1}{|}^{2}+|{\lambda }_{3}{|}^{2})}}{|HH\rangle }_{12}{(\frac{{\lambda }_{0}}{{\lambda }_{1}}|HH\rangle +|VV\rangle )}_{34}\\  &  & +\frac{{\lambda }_{3}^{2}}{\sqrt{{P}_{A1}^{o}(|{\lambda }_{1}{|}^{2}+|{\lambda }_{3}{|}^{2})}}{|VV\rangle }_{12}{(\frac{{\lambda }_{0}}{{\lambda }_{1}}|HH\rangle -|VV\rangle )}_{34}]\\  & \otimes  & (\frac{{\lambda }_{3}^{2}}{\sqrt{|{\lambda }_{1}{|}^{4}+|{\lambda }_{3}{|}^{4}}}{|H\rangle }_{1^{\prime\prime} }+\frac{{\lambda }_{1}^{2}}{\sqrt{|{\lambda }_{1}{|}^{4}+|{\lambda }_{3}{|}^{4}}}{|V\rangle }_{1^{\prime\prime}})\\  & = & \frac{{\lambda }_{1}^{2}{\lambda }_{3}^{2}}{\sqrt{{P}_{A1}^{o}(|{\lambda }_{1}{|}^{2}+|{\lambda }_{3}{|}^{2})(|{\lambda }_{1}{|}^{4}+|{\lambda }_{3}{|}^{4})}}[{|HHH\rangle }_{11^{\prime\prime} 2}\\  &  & \times {(\frac{{\lambda }_{0}}{{\lambda }_{1}}|HH\rangle +|VV\rangle )}_{34}+{|VVV\rangle }_{11^{\prime\prime} 2}{(\frac{{\lambda }_{0}}{{\lambda }_{1}}|HH\rangle -|VV\rangle )}_{34}]\\  &  & +\frac{{\lambda }_{1}^{4}{|HVH\rangle }_{11^{\prime\prime} 2}}{\sqrt{{P}_{A1}^{o}(|{\lambda }_{1}{|}^{2}+|{\lambda }_{3}{|}^{2})(|{\lambda }_{1}{|}^{4}+|{\lambda }_{3}{|}^{4})}}{(\frac{{\lambda }_{0}}{{\lambda }_{1}}|HH\rangle +|VV\rangle )}_{34}\\  &  & +\frac{{\lambda }_{3}^{4}}{\sqrt{{P}_{A1}^{o}(|{\lambda }_{1}{|}^{2}+|{\lambda }_{3}{|}^{2})(|{\lambda }_{1}{|}^{4}+|{\lambda }_{3}{|}^{4})}}\\  &  & \times {|VHV\rangle }_{11^{\prime\prime} 2}{(\frac{{\lambda }_{0}}{{\lambda }_{1}}|HH\rangle -|VV\rangle )}_{34}\mathrm{.}\end{array}\end{array}$$

Alice makes particle 1′′ and particle 1 go through the PCD, measures particle 1′′ with the basis {|+〉, |−〉} and operates *I* or *σ*_*Z*_ according to the measurement results of particle 1′′. If the output of PCD is even, the step is successful, otherwise the step fails, and Alice has to prepare the third ancillary particle and iterates above steps until the parity checking result is even. After two rounds, the probability of failure (i.e., the probability that the parity checking result is odd) is

18 $${P}_{A2}^{e}=\frac{|{\lambda }_{1}{|}^{8}+|{\lambda }_{3}{|}^{8}}{(|{\lambda }_{1}{|}^{2}+|{\lambda }_{3}{|}^{2})(|{\lambda }_{1}{|}^{4}+|{\lambda }_{3}{|}^{4})}({|\frac{{\lambda }_{0}}{{\lambda }_{1}}|}^{2}+1)\mathrm{.}$$

The success probability in the second round is

19 $${P}_{A2}^{e}=\frac{\mathrm{2|}{\lambda }_{1}{\lambda }_{3}{|}^{4}}{(|{\lambda }_{1}{|}^{2}+|{\lambda }_{3}{|}^{2})(|{\lambda }_{1}{|}^{4}+|{\lambda }_{3}{|}^{4})}({|\frac{{\lambda }_{0}}{{\lambda }_{1}}|}^{2}+1),$$

and the state is

20 $${|\varphi \rangle }_{1234}^{A2e}=\frac{1}{\sqrt{2({|\frac{{\lambda }_{0}}{{\lambda }_{1}}|}^{2}+1)}}[\frac{{\lambda }_{0}}{{\lambda }_{1}}{|HH\rangle }_{34}{(|HH\rangle +|VV\rangle )}_{12}+{|VV\rangle }_{34}{(|HH\rangle -|VV\rangle )}_{12}],$$

same as the state $|\varphi {\rangle }_{1234}^{A{\rm{1e}}}$. Hence, the success probability in the third round is

21 $${P}_{A3}^{e}=\frac{\mathrm{2|}{\lambda }_{1}{\lambda }_{3}{|}^{8}}{(|{\lambda }_{1}{|}^{2}+|{\lambda }_{3}{|}^{2})(|{\lambda }_{1}{|}^{4}+|{\lambda }_{3}{|}^{4})(|{\lambda }_{1}{|}^{8}+|{\lambda }_{3}{|}^{8})}(|\frac{{\lambda }_{0}}{{\lambda }_{1}}{|}^{2}+1),$$

and the success probability in the *m* th round is

22 $${P}_{Am}^{e}=\frac{\mathrm{2|}{\lambda }_{1}{\lambda }_{3}{|}^{{2}^{m}}}{\prod _{i\mathrm{=1}}^{m}({|{\lambda }_{1}|}^{{2}^{i}}+{|{\lambda }_{3}|}^{{2}^{i}})}({|\frac{{\lambda }_{0}}{{\lambda }_{1}}|}^{2}+1)$$

with the final state $|\varphi {\rangle }_{1234}^{Ame}$ which is same as the state $|\varphi {\rangle }_{1234}^{A{\rm{1e}}}$. After iterating the entanglement concentration process *m* rounds, the total success probability of Alice’s step is

23 $${P}_{A}=\sum _{i\mathrm{=1}}^{m}{P}_{Ai}^{e}\mathrm{.}$$

Because the state $|\varphi {\rangle }_{1234}^{Ame}$ after *m* rounds of Alice’s operations is same as $|\varphi {\rangle }_{1234}^{A{\rm{1e}}}$ of Eq. (14), here denote the system after Alice’s successful operations as $|\varphi {\rangle }_{1234}^{Ae}$, and Charlie does not need to know the number of rounds Alice operates.

After told that Alice’s steps are successful, Charlie continues to do the concentration. At the beginning, Charlie prepares an ancillary particle 3′ in the state

24 $${|\varphi \rangle }_{3^{\prime} }=\frac{{\lambda }_{1}}{\sqrt{|{\lambda }_{0}{|}^{2}+|{\lambda }_{1}{|}^{2}}}|H\rangle +\frac{{\lambda }_{0}}{\sqrt{|{\lambda }_{0}{|}^{2}+|{\lambda }_{1}{|}^{2}}}|V\rangle ,$$

makes particle 3 and particle 3′ go through PCD, measures particle 3′ with basis {|+〉, |−〉}, and operates *I* or *σ*_*Z*_ on particle 3, similar with Alice’s operations. According to the output of PCD, the final system is transformed from

25 $$\begin{array}{rcl}{|\varphi \rangle }_{1234}^{Ae}\otimes {|\varphi \rangle }_{3^{\prime} } & = & \frac{{\lambda }_{0}|{\lambda }_{1}|}{\sqrt{2}({|{\lambda }_{0}|}^{2}+{|{\lambda }_{1}|}^{2})}[{|HHH\rangle }_{33^{\prime} 4}{(|HH\rangle +|VV\rangle )}_{12}\\  &  & +{|VVV\rangle }_{33^{\prime} 4}{(|HH\rangle -|VV\rangle )}_{12}]+\frac{|{\lambda }_{1}|}{\sqrt{2}{\lambda }_{1}({|{\lambda }_{0}|}^{2}+{|{\lambda }_{1}|}^{2})}\\  &  & \times [{\lambda }_{0}^{2}{|HVH\rangle }_{33^{\prime} 4}{(|HH\rangle +|VV\rangle )}_{12}\\  &  & +{\lambda }_{1}^{2}{|VHV\rangle }_{33^{\prime} 4}{(|HH\rangle -|VV\rangle )}_{12}]\end{array}$$

into two classes,

26 $${|\varphi \rangle }_{1234}^{C1e}=\frac{1}{2}{(|HHHH\rangle +|HHVV\rangle +|VVHH\rangle -|VVVV\rangle )}_{1234}$$

with the probability

27 $${P}_{C1}^{e}=\frac{2{|{\lambda }_{0}{\lambda }_{1}|}^{2}}{{({|{\lambda }_{0}|}^{2}+{|{\lambda }_{1}|}^{2})}^{2}},$$

and

28 $${|\varphi \rangle }_{1234}^{C1o}=\frac{1}{\sqrt{2({|{\lambda }_{0}|}^{4}+{|{\lambda }_{1}|}^{4})}}[{\lambda }_{0}^{2}{|HH\rangle }_{34}{(|HH\rangle +|VV\rangle )}_{12}+{\lambda }_{1}^{2}{|VV\rangle }_{34}{(|HH\rangle -|VV\rangle )}_{12}]$$

with the probability ${P}_{C1}^{o}=\frac{{|{\lambda }_{0}|}^{4}+{|{\lambda }_{1}|}^{4}}{{({|{\lambda }_{0}|}^{2}+{|{\lambda }_{1}|}^{2})}^{2}}$. If the parity checking result is even, the system is in a perfect four-particle cluster state |*ψ*〉_1234_. If the parity checking result is odd, Charlie has to do another round to obtain the perfect four-particle cluster state. The success probability in the second round is

29 $${P}_{C2}^{e}={P}_{C1}^{o}\frac{\mathrm{2|}{\lambda }_{0}{|}^{4}|{\lambda }_{1}{|}^{4}}{{(|{\lambda }_{0}{|}^{4}+|{\lambda }_{1}{|}^{4})}^{2}}=\frac{\mathrm{2|}{\lambda }_{0}{|}^{4}|{\lambda }_{1}{|}^{4}}{(|{\lambda }_{0}{|}^{4}+|{\lambda }_{1}{|}^{4})(|{\lambda }_{0}{|}^{2}+|{\lambda }_{1}{|}^{2}{)}^{2}},$$

and the probability of failure in the second round is ${P}_{C2}^{o}=1-{P}_{C1}^{e}-{P}_{C2}^{e}$. The success probability in the *n*th round is

30 $${P}_{Cn}^{e}=\frac{2{|{\lambda }_{0}|}^{{2}^{n}}{|{\lambda }_{1}|}^{{2}^{n}}{P}_{C,n-1}^{o}}{{({|{\lambda }_{0}|}^{{2}^{n}}+{|{\lambda }_{1}|}^{{2}^{n}})}^{2}}\mathrm{.}$$

Therefore, by iterating the entanglement concentration process *n* rounds, the total success probability in Charlie’s steps is

31 $${P}_{C}=\sum _{i\mathrm{=1}}^{n}{P}_{Ci}^{e}\mathrm{.}$$

After Alice’s *m* rounds operations and Charlie’s *n* rounds operations, the probability that the final state is in perfect cluster state is

32 $$P={P}_{A}{P}_{C}=\sum _{i\mathrm{=1}}^{m}{P}_{Ai}^{e}\sum _{j\mathrm{=1}}^{n}{P}_{Cj}^{e},$$

which depends on the coefficients of the initial state |Ψ〉_1234_ and the numbers of iterations performed by Alice and Charlie.

## Discussion

**Figure 4:**
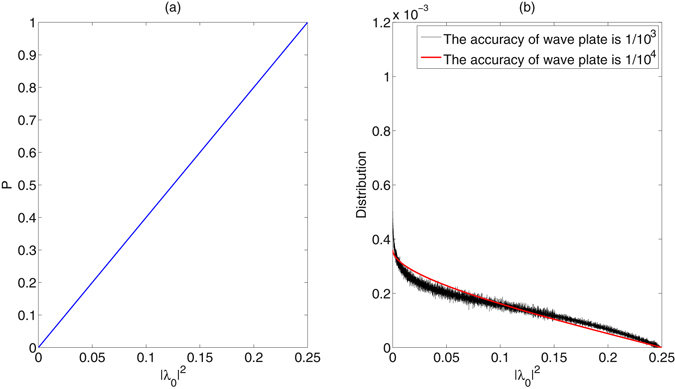
(**a**) The success probability of the ECP with linear optics vs the parameters |*λ*_0_|^2^ in the range [0,0.25]. (**b**) The distribution of the number of possible initial cluster states that can be concentrated by the ECP with linear optics vs the parameter |*λ*_0_|^2^ under different accuracies of the wave plate 1/10^3^ and 1/10^4^.

We introduce two ways to concentrate the entanglement from an arbitrary four-particle LCS |Ψ〉_1234_ = *λ*_0_|*HHHH*〉_1234_ + *λ*_1_|*HHVV*〉_1234_ + *λ*_2_|*VVHH*〉_1234_ + *λ*_3_|*VVVV*〉_1234_. The first ECP is realized by a series of PBSs and two rotate operations, and the success probability is 4|*λ*_0_|^2^ if the coefficients of |Ψ〉_1234_ satisfy *λ*_0_ *λ*_3_ = −*λ*_1_ *λ*_2_. The visible relationship between the success probability and the parameter |*λ*_0_|^2^ is shown in Fig. 4(a). Apparently, the success probability is 4 times the parameter |*λ*_0_|^2^. Furthermore, the wave plates are imperfect in experiment, so we discuss the affection of accuracy of the wave plates on the concentration. Ignored the global phases, we consider the number of possible initial cluster states that can be concentrated by the ECP with linear optics, and simulate the probability distributions of the number with the parameter |*λ*_0_|^2^ in Fig. 4(b) if the accuracies of the wave plate in Fig. 1 are 1/10^3^ and 1/10^4^. In Fig. 4(b), the number of initial states that can be concentrated by the ECP with linear optics decreases with |*λ*_0_|^2^ increasing. The higher the accuracy of the wave plates, the smoother the distribution of the number of the possible states.

Besides only using linear optics, another advantage of the ECP is that the scheme doesn’t need any ancillary particles. The only compromise is that the results need postselection. In Fig. 1, Bob and Charlie should observe that whether the detectors *D*_1_ and *D*_2_ click or not. Any detector clicks, the ECP with linear optics fails, else it succeeds. Thus the detection efficiency of the detectors in practice also affects that whether the ECP is successful or not. In Section II, we suppose the detection efficiency of both detectors *D*_1_ and *D*_2_ are 100%. However, the single-photon detectors are imperfect. The detection efficiency cannot reach 100%, and there exists dark counts in experiment. Therefore, more practical concentration of the entanglement for four-particle cluster states should be studied in the future.

The second ECP for four-particle LCSs is realized via cross-Kerr nonlinearity which can check the parity between the original particle and the ancillary particle. Compared with the first ECP protocol, two particles of the original state in the second ECP with cross-Kerr nonlinearity is reentered the devices again and again until the whole system is in a perfect cluster state. The iteration increases the final success probability, which is related with four parameters, the number of Alice’s iterations *m*, the number of Charlie’s iterations *n*, and the coefficients |*λ*_0_|^2^ and |*λ*_2_|^2^ of |Ψ〉_1234_. No matter how many iterations Alice does, the whole systems before Charlie operates are in the same states. Thus the number of Charlie’s iterations is independent with the number of Alice’s iterations.

**Figure 5:**
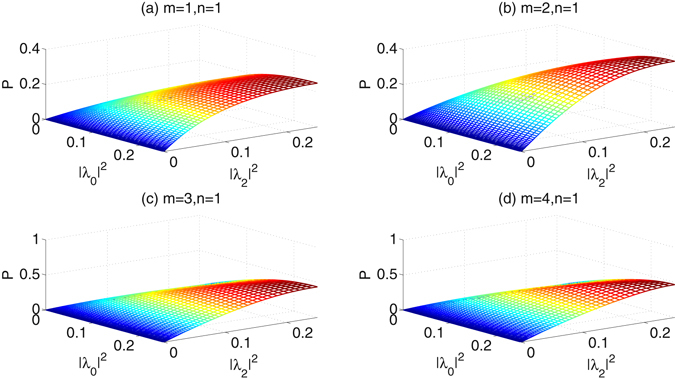
The success probabilities of the concentration in the second ECP vs the parameters |*λ*_0_|^2^ and |*λ*_2_|^2^, when the number of Charlie’s iterations is fixed as *n* = 1.

**Figure 6:**
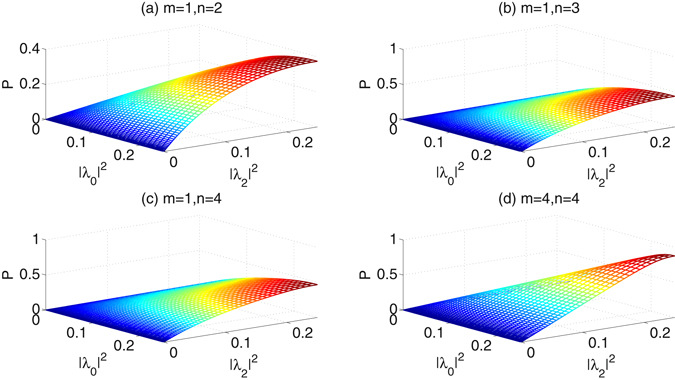
(**a–c**) The success probabilities of the concentration in the second ECP vs the parameters |*λ*_0_|^2^ and |*λ*_2_|^2^, when the number of Alice’s iterations is fixed as *m* = 1. (**d**) The success probabilities when both of *m* and *n* are equal to 4.

When the number of Charlie’s iterations is fixed as *n* = 1, the success probabilities as a function of the coefficients |*λ*_0_|^2^ and |*λ*_2_|^2^ are shown in Fig. 5. Figure 6(a–c) give the results when that of Alice’s iterations is fixed as *m* = 1, and Fig. 6(d) shows the success probabilities when both of *m* and *n* are equal to 4. According to the simulation, we obtain the following conclusions: (i) With the parameters |*λ*_0_|^2^ and |*λ*_2_|^2^ increasing, the success probabilities increase. (ii) Both of Alice’s iterations and Charlie’s iterations can efficiently increase the success probabilities. (iii) The influence degree of Alice’s iterations on the success probabilities is similar as that of Charlie’s iterations. (iv) After 4 Alice’s iterations and 4 Charlie’s iterations, the success probabilities would reach more than 90%.

Compared with the first ECP with linear optics in Fig. 4(a), the second ECP with cross-Kerr nonlinearity in Fig. 6(d), though it is more difficult to be realized, would reach higher success probabilities for the same parameter |*λ*_0_|^2^. Besides the iteration increasing the success probabilities, the reason is that the success probability of the first ECP with linear optics is *P* = 4|*λ*_0_|^2^ which should satisfy the hypotheses |*λ*_0_| ≤ |*λ*_1_| and |*λ*_0_| ≤ |*λ*_2_|. To sum up, we introduce two ECPs for four-particle LCSs, one with linear optics, the other with cross-Kerr nonlinearity. The first ECP is easily realized in experiment, while the success probability in the second one can reach more than 90% after 4 Alice’s iterations and 4 Charlie’s iterations. The wide application of cluster states makes our two ECPs play different important roles on quantum communication.

## Electronic supplementary material

Supplementary Material
