# Supplementary material for: Entanglement concentration for arbitrary four-particle linear cluster states
Source: Sci Rep. 2017 May 16;7:1982. doi: 10.1038/s41598-017-02146-9 (PMC5434068; doi:10.1038/s41598-017-02146-9)
Supplement: Supplementary file 1 — Supplementary Material [file 41598_2017_2146_MOESM1_ESM.pdf]

Entanglement concentration for arbitrary four-particle linear cluster states

Ting-Ting Song, Xiaoqing Tan, and Tianyin Wang

February 5, 2017

Supplementary Material

If Alice, Bob, Charlie and Daniel are only permitted to perform local operations on the particles hold by themselves, according to Eq.(3) of main text the state $|\Psi\rangle_{1234}$ will be changed into

$$\begin{aligned}
& L_1 \otimes L_2 \otimes L_3 \otimes L_4 |\Psi\rangle_{1234} \\
= & (\lambda_0 a_1 a_2 a_3 a_4 + \lambda_1 a_1 a_2 b_3 b_4 + \lambda_2 b_1 b_2 a_3 a_4 + \lambda_3 b_1 b_2 b_3 b_4)|HHHH\rangle \\
+ & (\lambda_0 a_1 a_2 a_3 c_4 + \lambda_1 a_1 a_2 b_3 d_4 + \lambda_2 b_1 b_2 a_3 c_4 + \lambda_3 b_1 b_2 b_3 d_4)|HHHV\rangle \\
+ & (\lambda_0 a_1 a_2 c_3 a_4 + \lambda_1 a_1 a_2 d_3 b_4 + \lambda_2 b_1 b_2 c_3 a_4 + \lambda_3 b_1 b_2 d_3 b_4)|HHVH\rangle \\
+ & (\lambda_0 a_1 a_2 c_3 c_4 + \lambda_1 a_1 a_2 d_3 d_4 + \lambda_2 b_1 b_2 c_3 c_4 + \lambda_3 b_1 b_2 d_3 d_4)|HHVV\rangle \\
+ & (\lambda_0 a_1 c_2 a_3 a_4 + \lambda_1 a_1 c_2 b_3 b_4 + \lambda_2 b_1 d_2 a_3 a_4 + \lambda_3 b_1 d_2 b_3 b_4)|HVHH\rangle \\
+ & (\lambda_0 a_1 c_2 a_3 c_4 + \lambda_1 a_1 c_2 b_3 d_4 + \lambda_2 b_1 d_2 a_3 c_4 + \lambda_3 b_1 d_2 b_3 d_4)|HVHV\rangle \\
+ & (\lambda_0 a_1 c_2 c_3 a_4 + \lambda_1 a_1 c_2 d_3 b_4 + \lambda_2 b_1 d_2 c_3 a_4 + \lambda_3 b_1 d_2 d_3 b_4)|HVVH\rangle \\
+ & (\lambda_0 a_1 c_2 c_3 c_4 + \lambda_1 a_1 c_2 d_3 d_4 + \lambda_2 b_1 d_2 c_3 c_4 + \lambda_3 b_1 d_2 d_3 d_4)|HVVV\rangle \\
+ & (\lambda_0 c_1 a_2 a_3 a_4 + \lambda_1 c_1 a_2 b_3 b_4 + \lambda_2 d_1 b_2 a_3 a_4 + \lambda_3 d_1 b_2 b_3 b_4)|VHHH\rangle \\
+ & (\lambda_0 c_1 a_2 a_3 c_4 + \lambda_1 c_1 a_2 b_3 d_4 + \lambda_2 d_1 b_2 a_3 c_4 + \lambda_3 d_1 b_2 b_3 d_4)|VHHV\rangle \\
+ & (\lambda_0 c_1 a_2 c_3 a_4 + \lambda_1 c_1 a_2 d_3 b_4 + \lambda_2 d_1 b_2 c_3 a_4 + \lambda_3 d_1 b_2 d_3 b_4)|VHVH\rangle \\
+ & (\lambda_0 c_1 a_2 c_3 c_4 + \lambda_1 c_1 a_2 d_3 d_4 + \lambda_2 d_1 b_2 c_3 c_4 + \lambda_3 d_1 b_2 d_3 d_4)|VHVV\rangle \\
+ & (\lambda_0 c_1 c_2 a_3 a_4 + \lambda_1 c_1 c_2 b_3 b_4 + \lambda_2 d_1 d_2 a_3 a_4 + \lambda_3 d_1 d_2 b_3 b_4)|VVHH\rangle \\
+ & (\lambda_0 c_1 c_2 a_3 c_4 + \lambda_1 c_1 c_2 b_3 d_4 + \lambda_2 d_1 d_2 a_3 c_4 + \lambda_3 d_1 d_2 b_3 d_4)|VVHV\rangle \\
+ & (\lambda_0 c_1 c_2 c_3 a_4 + \lambda_1 c_1 c_2 d_3 b_4 + \lambda_2 d_1 d_2 c_3 a_4 + \lambda_3 d_1 d_2 d_3 b_4)|VVVH\rangle \\
+ & (\lambda_0 c_1 c_2 c_3 c_4 + \lambda_1 c_1 c_2 d_3 d_4 + \lambda_2 d_1 d_2 c_3 c_4 + \lambda_3 d_1 d_2 d_3 d_4)|VVVV\rangle. \qquad (1)
\end{aligned}$$

The coefficients of terms in Eq.(1) should satisfy the basic conditions,

$$\begin{aligned}
& f_1 = f_4 = f_{13} = -f_{16} \neq 0, \\
& f_i = 0, i \in [1,16]_Z \backslash \{1,4,13,16\}, \qquad (2)
\end{aligned}$$

where the coefficient of ith term in Eq.(1) is denoted as f_i.

When there is no operation on particle 4, i.e. $L_4 = I = |H\rangle\langle H| + |V\rangle\langle V|$ which means $a_4 = d_4 = 1$ and $b_4 = c_4 = 0$ in Eq.(1), substitute $a_4 = d_4 = 1$ and $b_4 = c_4 = 0$ in Eq.(1), the system is without

normalized changed into

$$\begin{aligned}
& |\Psi'\rangle_{1234} \\
= \; & L_1 \otimes L_2 \otimes L_3 \otimes I|\Psi\rangle_{1234} \\
= \; & (\lambda_0 a_1 a_2 a_3 + \lambda_2 b_1 b_2 a_3)|HHHH\rangle + (\lambda_1 a_1 a_2 b_3 + \lambda_3 b_1 b_2 b_3)|HHHV\rangle \\
& +(\lambda_0 a_1 a_2 c_3 + \lambda_2 b_1 b_2 c_3)|HHVH\rangle + (\lambda_1 a_1 a_2 d_3 + \lambda_3 b_1 b_2 d_3)|HHVV\rangle \\
& +(\lambda_0 a_1 c_2 a_3 + \lambda_2 b_1 d_2 a_3)|HVHH\rangle + (\lambda_1 a_1 c_2 b_3 + \lambda_3 b_1 d_2 b_3)|HVHV\rangle \\
& +(\lambda_0 a_1 c_2 c_3 + \lambda_2 b_1 d_2 c_3)|HVVH\rangle + (\lambda_1 a_1 c_2 d_3 + \lambda_3 b_1 d_2 d_3)|HVVV\rangle \\
& +(\lambda_0 c_1 a_2 a_3 + \lambda_2 d_1 b_2 a_3)|VHHH\rangle + (\lambda_1 c_1 a_2 b_3 + \lambda_3 d_1 b_2 b_3)|VHHV\rangle \\
& +(\lambda_0 c_1 a_2 c_3 + \lambda_2 d_1 b_2 c_3)|VHVH\rangle + (\lambda_1 c_1 a_2 d_3 + \lambda_3 d_1 b_2 d_3)|VHVV\rangle \\
& +(\lambda_0 c_1 c_2 a_3 + \lambda_2 d_1 d_2 a_3)|VVHH\rangle + (\lambda_1 c_1 c_2 b_3 + \lambda_3 d_1 d_2 b_3)|VVHV\rangle \\
& +(\lambda_0 c_1 c_2 c_3 + \lambda_2 d_1 d_2 c_3)|VVVH\rangle + (\lambda_1 c_1 c_2 d_3 + \lambda_3 d_1 d_2 d_3)|VVVV\rangle.
\end{aligned} \tag{3}$$

If the coefficients in the last above equation are denoted as $f'(i), i \in [1, 16]_Z$, they satisfy the conditions in Eq.(5) of main text. Now we solve the relationship between λ_i and the coefficients of L_i.

Following

$$f_1' = (\lambda_0 a_1 a_2 + \lambda_2 b_1 b_2) a_3 \neq 0, \; f_3' = (\lambda_0 a_1 a_2 + \lambda_2 b_1 b_2) c_3 = 0, \tag{4}$$

we obtain

$$\begin{cases} c_3 = 0, \\ a_3 \neq 0, \\ \lambda_0 a_1 a_2 + \lambda_2 b_1 b_2 \neq 0. \end{cases} \tag{5}$$

Likewise, because of the formulas $f_{14}' = 0$ and $f_{16}' \neq 0$, there exists

$$\begin{cases} b_3 = 0, \\ d_3 \neq 0, \\ \lambda_1 c_1 c_2 + \lambda_3 d_1 d_2 \neq 0. \end{cases} \tag{6}$$

Following the results in Eqs.(5-6) and the equations $f_5' = f_8' = f_9' = f_{12}' = 0$, we obtain the relationship as

$$\begin{cases} \lambda_0 a_1 c_2 + \lambda_2 b_1 d_2 = 0, \\ \lambda_1 a_1 c_2 + \lambda_3 b_1 d_2 = 0, \\ \lambda_0 c_1 a_2 + \lambda_2 d_1 b_2 = 0, \\ \lambda_1 c_1 a_2 + \lambda_3 d_1 b_2 = 0. \end{cases} \tag{7}$$

Furthermore, based on $f_1' = f_4' = f_{13}' = -f_{16}' \neq 0$, the results of the parameters $a_i, b_i, c_i, d_i (i = 1, 2, 3)$ in Eq.(3) have three solutions.

The first solution is $\lambda_1 \lambda_2 - \lambda_0 \lambda_3 = 0$. The second solution is

$$\begin{cases} a_1 = d_1 = a_2 = d_2 = b_3 = c_3 = 0, \\ b_1 c_1 b_2 c_2 a_3 d_3 \neq 0, \\ \lambda_2 a_3 = \lambda_3 d_3, \\ \lambda_2 b_1 b_2 = \lambda_0 c_1 c_2, \\ \lambda_1 \lambda_2 + \lambda_0 \lambda_3 = 0, \end{cases} \tag{8}$$

with the final system

$$
\begin{aligned}
& |\Psi'^{(2)}\rangle_{1234} \\
= \; & \lambda_2 b_1 b_2 a_3 |HHHH\rangle + \lambda_3 b_1 b_2 d_3 |HHVV\rangle + \lambda_0 c_1 c_2 a_3 |VVHH\rangle + \lambda_1 c_1 c_2 d_3 |VVVV\rangle \\
= \; & \lambda_2 b_1 b_2 a_3 (|HHHH\rangle + |HHVV\rangle + |VVHH\rangle - |VVVV\rangle). \qquad (9)
\end{aligned}
$$

The third solution is

$$
\begin{cases}
b_1 = c_1 = b_2 = c_2 = b_3 = c_3 = 0, \\
a_1 d_1 a_2 d_2 a_3 d_3 \neq 0, \\
\lambda_2 a_3 + \lambda_3 d_3 = 0, \\
\lambda_1 a_1 a_2 + \lambda_3 d_1 d_2 = 0, \\
\lambda_1 \lambda_2 + \lambda_0 \lambda_3 = 0,
\end{cases} \qquad (10)
$$

with the final state

$$
\begin{aligned}
& |\Psi'^{(3)}\rangle_{1234} \\
= \; & \lambda_0 a_1 a_2 a_3 |HHHH\rangle + \lambda_1 a_1 a_2 d_3 |HHVV\rangle + \lambda_2 d_1 d_2 a_3 |VVHH\rangle + \lambda_3 d_1 d_2 d_3 |VVVV\rangle \\
= \; & \lambda_2 d_1 d_2 a_3 (|HHHH\rangle + |HHVV\rangle + |VVHH\rangle - |VVVV\rangle). \qquad (11)
\end{aligned}
$$
